# Therapeutic potential of dental follicle-derived stem cells and gold nanoparticles on osteoarthritic temporomandibular joint in guinea pigs (randomized prospective study)

**DOI:** 10.1186/s12903-026-09529-3

**Published:** 2026-08-15

**Authors:** Amira Mohsen Elsherbini, Samah Khaled Ezzat

**Affiliations:** 1 Faculty of Dentistry, Mansoura University, Mansoura, Egypt; 2 Faculty of Dentistry, New Mansoura University, New Mansoura, Egypt

**Keywords:** Temporomandibular joint, Osteoarthritis, Complete Freund's Adjuvant, Dental follicle stem cells, Gold nanoparticles

## Abstract

**Background:**

Osteoarthritis (OA) is a complicated autoimmune disease that often causes invasive inflammatory infiltration and serious articular destruction. The global prevalence of temporomandibular disorders (TMDs) is estimated to be around 30% of the population. Several clinical drugs have been reported to have adverse effects ranging from increased inflammation to liver cirrhosis. Even with the introduction of gene therapy, there is still a growing need for therapeutic alternatives for OA. This study aimed to evaluate the effect of dental follicle-derived stem cells (DFSCs) versus gold nanoparticles (AuNPs), on temporomandibular joint (TMJ) osteoarthritis induced by Complete Freund’s Adjuvant (CFA).

**Methods:**

In this study, *for osteoarthritis induction*,* twice intra-articular injections of complete Freund’s* adjuvant (CFA) were performed, once at the beginning of the experiment and again after two weeks. After 4 weeks, the osteoarthritis animals were randomly allocated into three equal groups: osteoarthritis group, osteoarthritis group treated with Nanogold (AuNPs), and osteoarthritis group treated with Dental follicle stem cells (DFSCs). After one month, the TMJs were fixed and processed for staining with hematoxylin and eosin, and the disc thickness was assessed; results were statistically evaluated.

**Results:**

The osteoarthritis group showed erosion of cartilage and subchondral bone and a thickened disc. Both DFSCs and AuNPs reduced the inflammatory signs. Though statistically non-significant, DFSCs showed a greater reduction in disc thickness.

**Conclusion:**

This study’s results highlighted the potential use of DFSCs and AuNPs as a promising treatment in TMJ osteoarthritis.

**Supplementary Information:**

The online version contains supplementary material available at 10.1186/s12903-026-09529-3.

## Background

Temporomandibular joint disorders (TMD), such as osteoarthritis and rheumatoid arthritis, are disabling disorders that impair masticatory system functions. Limited jaw movement, pain, malocclusion, joint clicking, and clenching are the main symptoms associated with TMD that dramatically affect the patients’ quality of life. The damage that affects joint structures, such as bone destruction and internal disc derangement, is attributed to the patient’s inflammatory response [1].

Complete Freund’s adjuvant (CFA) has been used to induce arthritis for physiological, biochemical, and histopathological studies [2]. CFA is composed of a solution of dried inactivated mycobacteria and antigen emulsified in mineral oil. After its injection into the joint intra-articular space, pain and edema appear within 12 h. Afterwards, destruction in bone and articular cartilage proceeds and may persist for several weeks [2, 3].

Proinflammatory cytokines such as TNF-α, IL-1β, IL-6, and IL-8 are highly expressed in the serum and synovial fluid of CFA-induced joint arthritis models. This expression is mainly attributed to disturbances in bone and articular cartilage formation [3, 4].

Stem cells derived from the ectomesenchyme of the dental follicle (DFSCs) enclosing the developing tooth germ before eruption are considered progenitor cells for cementoblasts, periodontal ligament cells, and osteoblasts. These cells are mainly obtained from the dental follicle of human third molars, possess proliferative capacity, and express cell surface antigens like those of other dental stem cells [5]. DFSCs express Notch1 and Nestin stem cell markers and have the capacity to produce periodontium tissues, including alveolar bone, periodontal ligament (PDL), and cementum. Moreover, they were capable of hard tissue formation both in vitro and in vivo [5]. DFSCs show osteogenic differentiation capacity when cultured under appropriate conditions. Also, DFSCs may differentiate into cementoblast-like cells when cultured with BMP-2/-7 and enamel matrix derivatives [6]. Moreover, DFSCs showed the ability to differentiate into chondrocytes and osteoblasts that may be crucial in TMJ regeneration [7]. Studies revealed that DFSCs have a vital role in the treatment of inflammatory and autoimmune diseases in animal models; they also showed the ability to form salivary gland acinar and ductal cells [8].

*Nanoscale therapeutic or diagnostic devices and nanoparticles have been introduced for the detection and treatment of arthritis in its early stages* [9]. *Gold nanoparticles (AuNPs*) was reported to hinder the progression of collagen-induced arthritis (CIA) in rats. Several studies showed that AuNPs are potential antioxidants, reducing reactive oxygen species, including H2O2 and the superoxide anion radical (O·̄2) [10].

TMJ osteoarthritis is a chronic degenerative disease that leads to cartilage destruction, subchondral bone changes, and impaired mandibular function. Although several beneficial effects have been reported, the regenerative potential of therapies such as dental follicle stem cells and gold nanoparticles has yet to be fully elucidated. Herein, this study investigated the potential role of dental follicle stem cells and gold nanoparticles in alleviating TMJ osteoarthritic changes in guinea pigs. The null hypothesis was that there would be no significant difference in articular disc thickness between the osteoarthritis control group and the two treatment groups.

## Materials and methods

### Aim

To investigate the possible role of dental follicle stem cells and gold nanoparticles in alleviating TMJ osteoarthritic changes in guinea pigs.

### In vitro

#### Source and characterization of Dental follicle stem cells

A dental follicle stem cell line was purchased from Nile Research Center (Mansoura, Egypt). Thawing of cryopreserved cells was done under proper aseptic technique, by inserting the lower half of the cryovial with the frozen cells for 60 s in a 37 °C water bath.

The cryovial was decontaminated with 70% ethanol. Using a sterile transfer pipette, cells were gently resuspended and transferred to a sterile 15-mL conical tube containing pre-warmed 5 mL DMEM with 10% fetal-calf serum (FCS), at 37 °C. All steps were performed in sterilized conditions. The DFSCs were centrifuged for 3 min at 200×g, and the supernatant was discarded while the cell pellet was resuspended in fresh, pre-warmed DMEM and transferred to a T25 flask, then incubated in a humidified incubator at 37 °C with 5% CO2 [10].

### Characterization of DFSCs

Briefly, Cells were incubated with 10*µ*L of primary antibody (1:100 ratio) at 4 °C for 45 min.

Fluorochrome-conjugated monoclonal antibodies against CD14 and CD34 (hematopoietic stem cell marker) and CD44, CD90 and CD73(mesenchymal stem cell marker) were used (ThermoFisher SCIENTIFIC, USA). The cells were centrifuged at 12,000 rpm for 15 min and washed with flow cytometry washing buffer and secondary antibodies, including Goat anti-mouse IgG-FITC (*F5262*, Sigma-Aldrich, USA) and Goat anti-mouse IgM-FITC (ab97229, Abcam, USA) were added, and the tubes were incubated at 4 °C in the dark for 45 min. After a washing step, the cells were analyzed by a flow cytometry analyzer (BD FACS Calibur) [11].

#### Source and characterization of gold nanoparticles (AuNPs)

Gold nanoparticle suspension was purchased ready-made from Nanogate company (9254 Huda Shaarawy St, Al Abageyah, Mokattam, Cairo Governorate 11571, Egypt). As reported by the manufacturer gold nanoparticle suspension was prepared by the chemical reduction method, as stated by Herizchi et al., 2016 [12]. The manufactured gold nanoparticle suspension was tested for optical properties, and UV-V absorption spectra were obtained on an Ocean Optics USB2000 + VIS-NIR Fiber optic spectrophotometer. The size and shape of the gold nanoparticles were tested by transmission electron microscope (TEM) JEOL JEM-2100 high-resolution TEM at an accelerating voltage of 200 kV [13].

### Study design

**Fig. 1:**
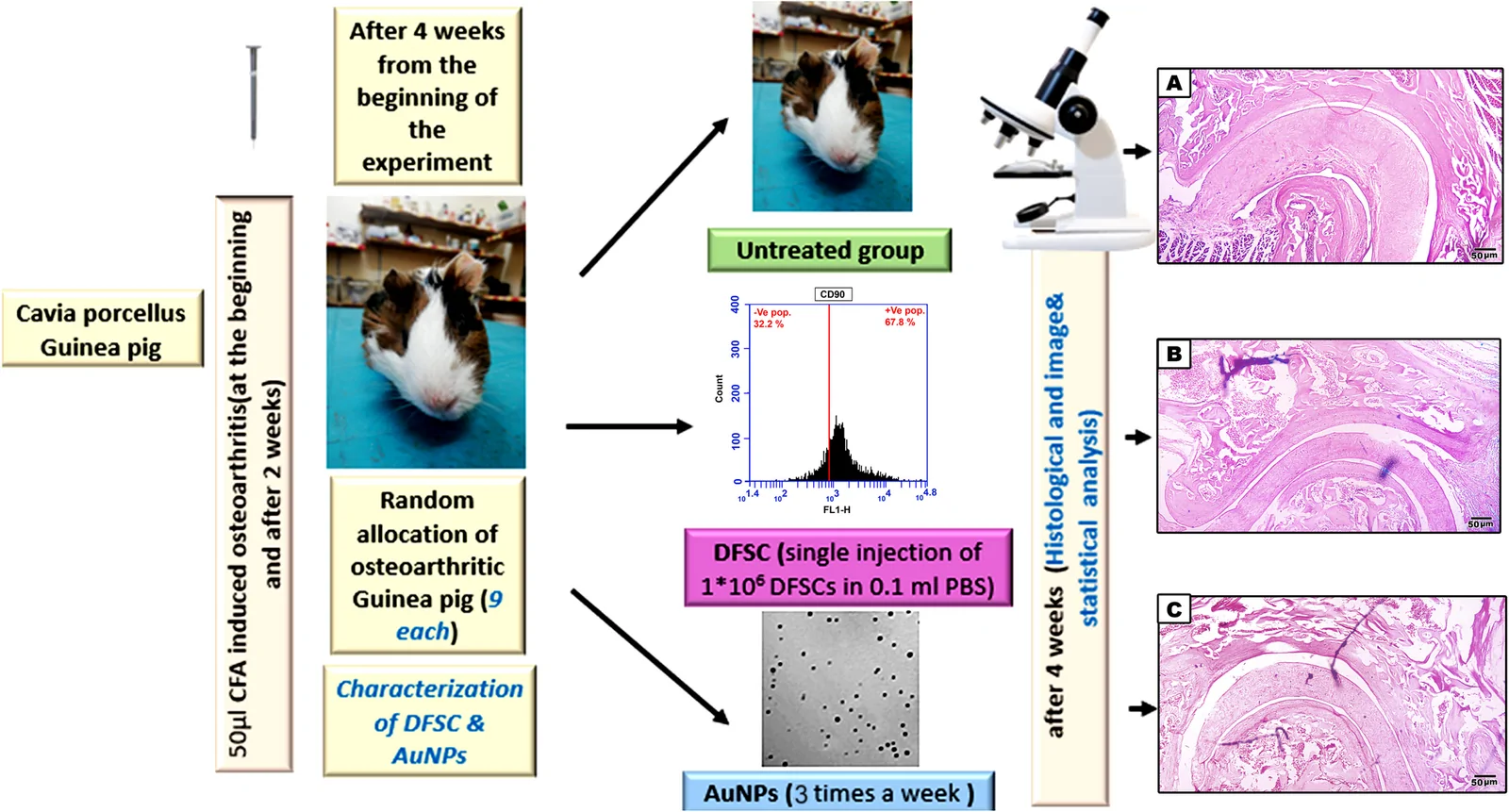
Graphical abstract

The guinea pigs got free access to a sterilized diet and water. All experimental procedures were standardized and approved by the ethical committee, Faculty of Dentistry, Mansoura University, Egypt (No: A22020822) and complied with ARRIVE reporting guidelines. All procedures were in accordance with the American Veterinary Medical Association guidelines. A priori sample size was calculated using G*Power 3.1.9.2. A sample size of 27 guinea pigs was used. The F test was used (ANOVA: fixed effects, omnibus, one way) with a 0.05 alpha significance level and 0.8 power to achieve a large effect size of 0.7. (Fig. 1)

### Animal grouping and osteoarthritis induction

The potential confounding variables in this study were weight, gender, and age. Inclusion criteria: Only males, pathogen-free, no prior TMJ problems, not subjected to any treatment or used in another experimental design. Exclusion criteria: underweight or aged animals. Females were excluded to avoid the effect of hormonal changes. After osteoarthritis induction, severe ulceration or external abscess formation was considered a humane endpoint; any mortality or samples damaged during the histological process were excluded. Twenty-seven adult, male Cavia porcellus guinea pigs, aged 4–6 months, weighing 250 –350 g (purchased from *VACSERA - EGY VEC*, Egypt), were acclimatized and housed in individual cages with free access to food under standardized conditions. They were kept in a 12:12 h light-dark cycle-controlled room with a temperature of 22 °C, and relative humidity between 65 and 70%. *All animals were provided and professionally handled by expert personnel from the Global Center for Experimental Research (Mansoura*,* Egypt).*

Osteoarthritis was induced by an invasive model through intra-articular injection into the right TMJ with 50 µl of CFA (Sigma-Aldrich, Germany) twice at the beginning of the experiment and after two weeks. Clinical osteoarthritis development was evaluated by a blinded examiner every two days [14, 15]. After 4 weeks, osteoarthritic animals were allocated randomly, using a random-number table, into 3 equal groups; osteoarthritis group, osteoarthritis treated with Dental follicle stem cells (DFSCs) group and osteoarthritis treated with Nanogold (AuNPs) group. Single injection of 1*10^6^ DFSCs suspended in 0.1 ml of PBS was locally injected in osteoarthritic TMJs, in DFSCs treated group. AuNPs were injected intra-articularly 3 times per week (the initial concentration of AuNPs was 50 µg/mL) for four weeks in AuNPs treated group [16]. All procedures, including induction, therapeutic administration, monitoring, and euthanasia were conducted in the morning to avoid the potential effect of circadian rhythm.

### Biopsy collection

After 1 month of treatment, guinea pigs were euthanized humanely, according to AVMA Guidelines for the euthanasia of animals [17], using intraperitoneal injection with 5 mg/kg xylazine, (Xylaject, ADWIA Co., Cairo, Egypt)) and 40 mg/kg ketamine (ketamine hydrochloride, Panpharma GmbH, Germany) for anesthesia to decrease stress and pain, followed by intraperitoneal anesthesia with 1% sodium pentobarbital at 300 mg/kg. Osteoarthritic right TMJs were harvested in buffered formalin, decalcified, and processed for histological examination by routine Hematoxylin and Eosin (H&E). The disc thickness was measured, and the data were statistically analyzed.

### Image analysis and statistical analysis

Images were captured using Olympus^®^ digital camera mounted on an Olympus^®^microscope (CX23, Wuhan Aliroad Medical Equipment Co., Ltd, Hubei, China). For measurements of the articular disc thickness, 2 readings for each zone (anterior band, intermediate zone, and posterior band) were recorded for each specimen. The sample size was reduced from 9 to 6 in each group due to unavoidable sample loss during processing. Data were tested for normality using the Shapiro-Wilk test. Normally distributed data were coded, tabulated, and analyzed using GraphPad Prism 9. Data were presented in mean and standard deviation (± SD). The effect of potential confounding variables was controlled via randomization and standardization of the experimental conditions. One-way analysis of variance (ANOVA) followed by a post-hoc Tukey test was used. A P value less than 0.05 was considered statistically significant.

## Results and discussion

## Results

### Characterization of gold nanoparticles and DFSCs

**Fig. 2:**
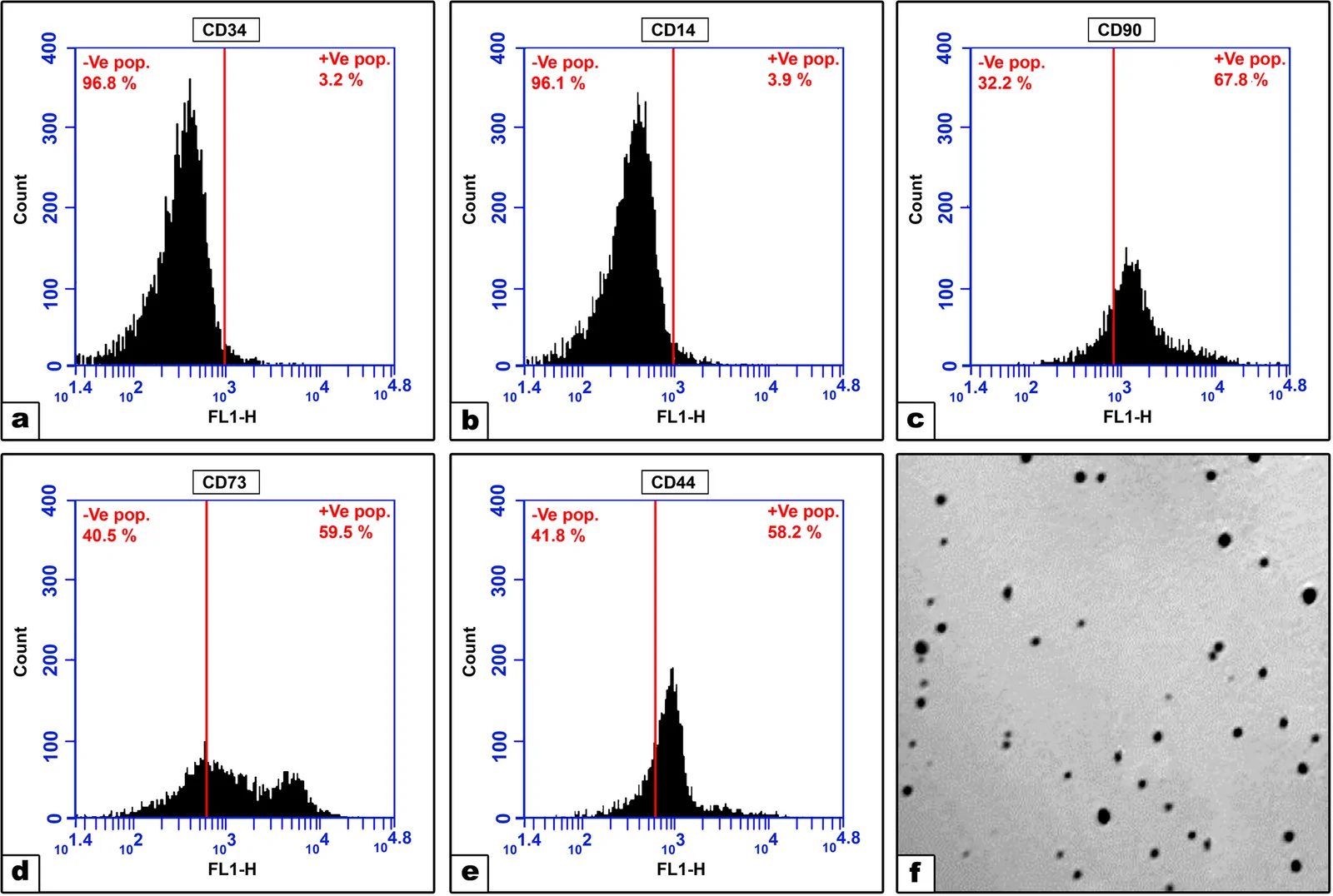
Representative flow cytometry analysis of DFSC’s surface markers showed negative expression for hematopoietic cell surface markers CD14 and CD34 and positive expression of mesenchymal cell surface markers CD90, CD73, and CD44 (**a-e**). **f**: Transmission electron microscope showing the shape and the particle size of AuNPs

DFSCs showed negative expression for hematopoietic cell surface markers CD14 and CD34 in addition to positive expression of mesenchymal cell surface markers CD90, CD73, and CD44 (Fig. 2a_e). The nanogold particles showed a spherical outline of an average size of 10 ± 2 nm and an optical property of (Abs.) λmax = 50 nm (Fig. 2f).

### Histological and image analysis results (H&E)

**Fig. 3:**
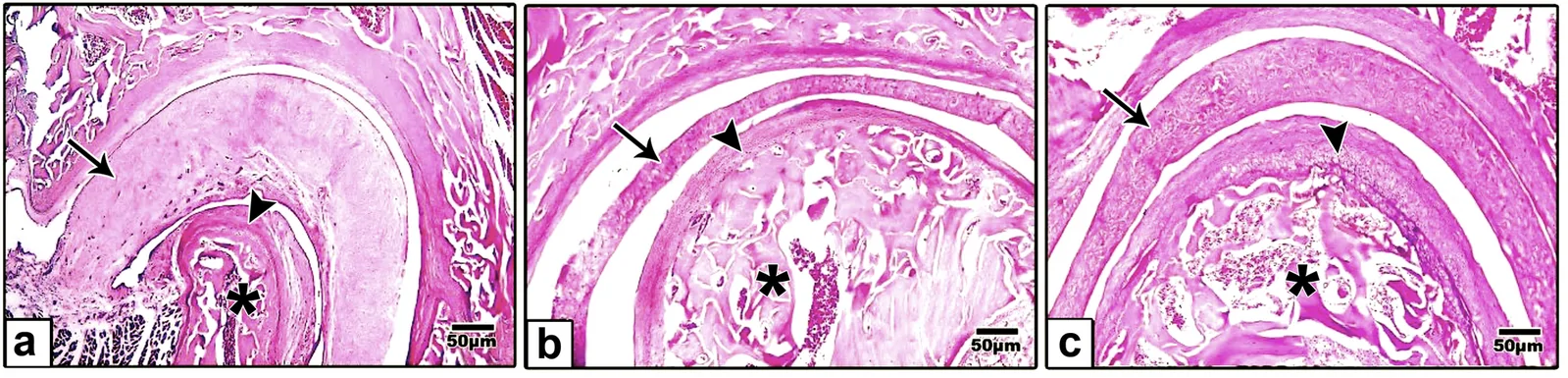
**A** photograph of H&E-stained TMJ sections showing osteoarthritic changes in the osteoarthritis group (**A**) and marked reduction in disc thickness and regain of subchondral bone and cartilage in both AuNPs group (**B**) and DFSCs group (**C**) (arrow…articular disc, arrowhead… hyaline cartilage, asterisk…condylar bone) (H&E, 50 μm)

**Fig. 4:**
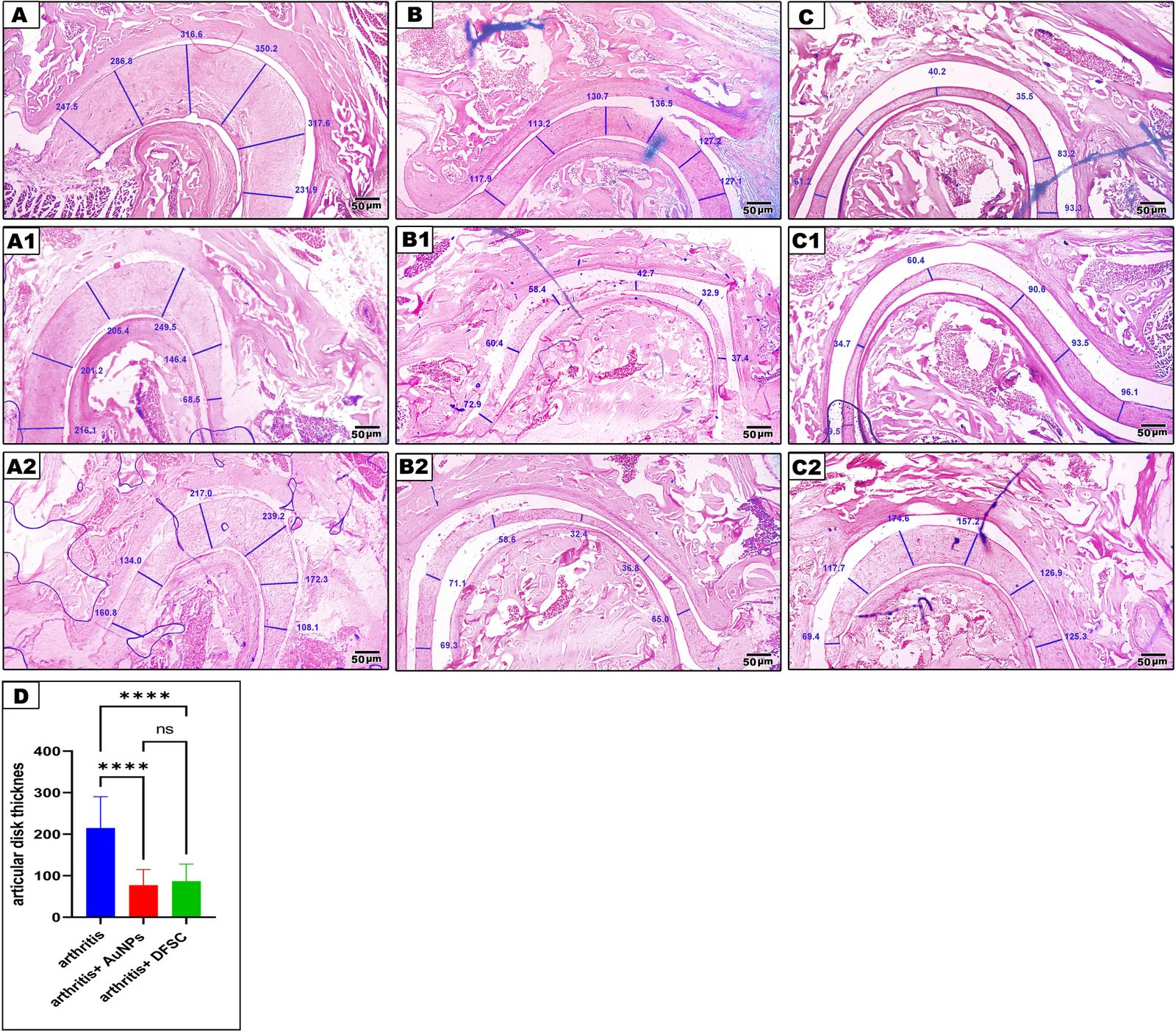
**A** photograph of articular disc thickness measurements in three different representative samples in each group osteoarthritis group (**A**, **A1**, **A2**), AuNPs group (**B**, **B1**, **B2**), and DFSCs group (**C**, **C1**, **C2**) (H&E, 50 μm). Statistical analysis of the articular disc thickness between the studied groups. One-way analysis of variance (ANOVA) followed by post-hoc Tukey test was used. A P value less than 0.05 was considered statistically significant. (Ns: non-significant) **D**

Osteoarthritis was confirmed clinically through swelling of the side of the intervention (Fig. 1), followed by histological assessment. The H&E results showed increased disc thickness and destructed subchondral bone, and cartilage marked with osteoclastic activity in the osteoarthritis group, in addition to increased thickness of the fibrocartilage layer. While both DFSCs and AuNPs treated groups showed reduced inflammatory signs, including reduced disc thickness and repair of subchondral bone and cartilage (Fig. 3). Statistically, there was no significant difference between the DFSCs and AuNPs treated groups, whereas the thickness of the articular disc in the osteoarthritis group was significantly larger than both the DFSCs and AuNPs treated groups (P value < 0.05) (Fig. 4).

## Discussion

Temporomandibular joint osteoarthritis (TMJOA) is a complex interplay of inflammatory, immune, and genetic factors. The pathophysiological process triggering TMJOA involves abnormal activation of different immune cells, which produce proinflammatory mediators such as cytokines, proteinases, and growth factors that mediate subsequent inflammatory responses, joint destruction, and several systemic complications [15, 18].

Utilization of adjuvant-induced Arthritis in different experimental models has been documented for studying the pathogenesis of osteoarthritic changes, with different protocols [15, 19, 20]. In this study, two intraarticular injections of 50 µg CFA in 50 µl on day 1 of the experiment were given to all animals to induce persistent inflammation and at day 14 to induce osteoarthritis [21]. Wang et al., 2017 reported that CFA injection increased iNOS, IL-1b, collagen type I, and aggrecan [22]. In addition, it was found that the increase of IL-1 expression has up-regulated Wnt-5 A, leading to MMP-3 expression via the JNK pathway in TMJ condylar chondrocytes of the rabbit [23].

In our results, the osteoarthritis group showed typical signs of TMJOA with marked swelling. Concomitantly, *Al-Mobireek* reported disc enlargement as well as condylar erosion and flattening in the osteoarthritic model of Guinea pigs [24]. Both DFSCs and AuNPs treated groups significantly alleviated the changes at the histological level with a reduction in bone/cartilage damage. During the study period, there were no physical changes like a change in fur color, and survival, indicating no systemic toxicological effect of both DFSCs and AuNPs, while there was a marked reduction of the local swelling compared with the osteoarthritis group.

Mesenchymal stem cells have gained immense interest as a potential TMJ therapy [25]. Among the proposed mechanisms of mesenchymal stem cells (MSCs) based therapies in TMJ cartilage/osteochondral defects and osteoarthritis treatment are improving cellular proliferation, migration, along with matrix synthesis and immune reactivity modulation [26].

Remarkably, dental tissue-derived MSCs similarly show therapeutic properties on TMJ-OA [27]. DFSCs are capable of multipotent differentiation with high pluripotency thanks to a series of pluripotent genes, including Octamer-binding transcription factor 4 (OCT-4), sex determining region Y box-2 (SOX-2) and NANOG, which can enable them to differentiate into osteoblasts, adipocytes, chondrocytes, cementoblasts and periodontal ligament cells as well as neuronal cells. Therefore, DFSCs are regarded as a promising candidate for regenerative medicine and tissue engineering [28].

Moreover, MSCs were reported to secrete a wide range of soluble factors, including macrovesicles and small extracellular vesicles that aid in the regulation of tissue microenvironment and in orchestrating the regenerative processes [29]. DFSCs-extracellular vesicles attenuated TMJ cartilage damage both in vitro as well as in vivo, via decreasing the downstream regulators of HIF-2*α*; VEGF and MMP13 [30]. Intra-articular injection of DFSCs extracellular vesicles regulates the local immune response via suppression of the metalloproteinases’ expression, including MMP3, MMP13, and MMP9, and increasing COL2 and SOX9 expression and TGF-beta release [31]. Thus, the improved outcomes of DFSCs articular injection may be explained by MSCs’ multilineage differentiation potential, which includes chondrogenic differentiation as well as osteogenic differentiation [26]. Moreover, Zibandeh et al. proposed an immunomodulatory role of DFSCs in arthritis via reduction of TNF-α secretion and upregulation of IL-10 secretion [32]. Additionally, DFSCs have demonstrated a potent anti-inflammatory effect as miRNA-146a-5p from DFSC-exosomes binds to the Traf6 gene, suppressing the downstream NF-κB signaling pathway on IL-1β-exposed mandibular condylar chondrocytes [33].

Nanomedicine has emerged as a new therapeutic strategy to effectively deliver drugs in inflamed joints for osteoarthritis treatment [34]. AuNPs as a promising metal nanoparticle have been utilized in different treatment strategies, including metallodrugs, immunotherapy, photothermal therapy, and synergistic therapy [35]. In this study, we used nanogold particles with an average size of 10 ± 2 nm and an optical property of (Abs.) λmax = 50 nm. *Abdel-Hakem et al.* studied three sizes of AuNPs (5, 25,70 nm) in a collagen-induced osteoarthritis model and revealed that AuNPs 25 nm elicited the maximum reduction of osteoarthritis severity [34]. Nanoparticles provide a controlled drug release, prolonged retention time, as well as enhanced penetration into TMJ tissue, thus increasing the efficacy of intra-articular injections. In addition, intra-articular injections increase bioavailability and reduce systemic adverse reactions [36–38].

AuNPs treated group showed marked improvement of the histological features of subchondral bone and cartilage with marked reduction of the articular disc thickness. Consistent with our results, Kirdaite et al. revealed an antioxidant effect of AuNPs via elevating the catalase activity level in the early stage of osteoarthritis [39]. Of note, inhibition of lipopolysaccharide -induced proinflammatory mediators was proposed in type II collagen-induced RA. Anti-inflammatory property of nanogold against osteoarthritis may be explained by modulating NF-κB pathways via suppressing COX-2 activity [40] and inhibition of MMPs without inducing cytotoxic effects [41]. Another study stated that nanogold decreased angiogenesis, macrophage infiltration, and proinflammatory cytokines as TNFα and IL-1b, in the synovium in a model of collagen induced arthritis in rats [42].

Furthermore, Pavlovich and Volkova, 2017 reported an anti-oxidant action of AuNPs, that led to suppression of joint swelling, synovial angiogenesis, and cartilage erosion in the initial stage of arthritis, without necrosis or apoptosis in fibroblast cells [41]. Collectively, the anti-inflammatory, and antiangiogenic effects of AuNP treatment may be responsible for improved osteoarthritic condition [43]. In contrast, Pascarelli et al.re reported that AuNPs stimulated expression of MMP1, MMP3, and MMP13 mRNA at 160 µM concentration on cultured human osteoarthritic chondrocytes via upregulation of iNOS production, and nitric oxide formation that led to chondrocytes apoptosis [44]. Contradictory results in similar experiments can be due to variable study conditions such as the particles’ physicochemical properties and different concentrations, along with the exposure duration and other parameters. The recent data on gold as a noble metal assigned it as a safe treatment of autoimmune inflammatory conditions [45]. AuNPs might affect the immune cells through macrophage modulation and thus regulating the inflammatory microenvironment and regenerative cytokine production. This mechanism has been explored as a potential therapeutic strategy for AuNPs in periodontal regeneration and prevention of periodontitis progression using 45 nm AuNPs [46].

### limitations

Though gold nanoparticles were reported to be safe and nontoxic [35], the lack of AuNPs toxicity assessment is one of the limitations of this study. Additionally, using a larger sample size, different concentrations, and longer periods of follow-up should be considered for proper evaluation of the therapeutic modalities.

## Conclusion

The current results showed possible therapeutic properties of both dental follicle stem cells and gold nanoparticles in the treatment of TMJ osteoarthritis; future studies are essential for elaborating the mechanism of action of both modalities and the possible effect of their combination.

## Supplementary Information

Supplementary Material 1.

## Data Availability

All data generated or analyzed during this study are included in this published article.

## Abbreviations

RA: Rheumatoid arthritis
TMD: Temporomandibular joint disorders
DFSCs: Dental follicle-derived stem cells
CFA: Complete Freund’s Adjuvant
AuNPs: Nanogold, Gold nanoparticles
TMJ: Temporomandibular joint
TNF-α: Tumor Necrosis Factor-alpha
IL-1b: Interleukin-1 beta
IL-6: Interleukin 6
IL-8: Interleukin 8
BMP-2/-7: Bone Morphogenic proteins
CIA: Collagen-induced arthritis
O·̄2: Superoxide anion radical
H2O2: Hydrogen peroxide
DMEM: Dulbecco’s Modified Eagle Medium
FCS: Fetal-calf serum
TEM: Transmission electron microscope
PBS: Phosphate buffer saline
TMJOA: Temporomandibular joint osteoarthritis
MSCs: Mesenchymal stem cells
HIF-2α: Hypoxia-inducible factor-2α
MMP13: Matrix metalloproteinases 13
VEGF: Vascular endothelial growth factor
NF-κB: Nuclear factor-kappa B
COX-2: Cyclooxygenase-2
iNOS: Inducible nitric oxide synthase
